# Treatment of Pulpless Teeth and Manner of Filling Roots

**Published:** 1881-12

**Authors:** N. W. Williams

**Affiliations:** Geneva, Switzerland


### THE DENTAL REGISTER.

VOL. XXXV.] DECEMBER, 1881. [No. 12.

### TREATMENT OF PULPLESS TEETH AND MANNER OF FILLING ROOTS.

BY N. W. WILLIAMS, D. D. S., GENEVA, SWITZERLAND.

*Read before the American Dental Association of Europe at Weisbaden, Aug.* 12, 1881.

The treatment of dead teeth and filling of roots is much better understood by the profession at present than formerly; and yet there is much ignorance shown on the subject by the great mass who are practicing dentistry. In the memory of most of us, a dead tooth was thought to be a fit subject for the forceps; at the present day the dentist who can not save a large per cent. of such teeth may be considered as being years behind the times. In the last few years a class of dentists has sprung up, which has made the wonderful discovery that the proper method of treating diseased teeth is to wrench them from the mouth, treat and fill the roots, and return them to their places. If the operation succeeds, it is imagined to be a wonderful thing, and as such is impressed with more than due emphasis upon the mind of the patient; at the same time the dentist who has the proper knowledge of the treatment of such teeth will succeed in saving them without making his patient suffer the pain and run the risk attending such an operation. Another ingenious method has been proclaimed of treating these unfortunate members; it consists of a new system of sewerage, by placing a tube in each root connecting with the cess-pool at the end, enabling the patient at will to draw out some of the sweet stuffs that may accumulate from day to day. I saw one of these cases in which the tube had become loose and had fallen out, the patient called to have it replaced ; I suggested the propriety of treating and filling the root, but the lady objected, as her dentist had told her never to have the root filled, as she would be subject to alveolar abscess ; she said when she felt pain or soreness, which was frequent, all she had to do was to suck and suck until she brought blood, and then it was all right.

In the treatment of dead teeth it is well to avoid one error which many of us, unwittingly, have fallen into, *i. e.*, overtreatment of them. When a nerve has died under a filling, or from any other cause, open the cavity so as to obtain ready access to each root, then carefully remove everything from them with small well tempered broaches, being very careful not to push any of the dead nerve or other debris beyond the point ; should a particle of this poisonous matter be pressed through the foramen, we may expect trouble, which may result in an alveolar abscess. Wash the roots throughly by winding small shreds of cotton around a small broach dipped in pure alcohol until there is no further discoloration of the cotton, and there remains no disagreeable odor. If the debris in the roots be dry no further treatment will be required and the roots should be filled immediately. But if there is a discharge of pus it is better to treat for a few days ; at each dressing wash thoroughly with the alcohol ; also, if the tooth is tender and inclined to periostitis, it is well to leave it open for a few days, after washing with the alcohol, without any dressing in the roots. This mode of treatment I have found will succeed in almost every case. When it fails it is very evident that some part of the treatment is at fault.

If there be a fistulous opening from the abscess at the point of the root, force carbolic acid through the root until it shows in the fistula, then fill the root immediately. The best material I have found to give the largest percentage of success in filling the roots of dead teeth is the oxychloride of zinc. I mix it to the consistency of cream, introducing it by winding shreds of cotton round a broach dipping or rolling it in the oxychloride until the cotton is thorougly saturated, then passing it to the point, packing it thorougly until the root is filled, leaving the cotton imbeded in the filling. With this material one is more likely to succeed in filling the root, for, the material being in liquid form, with the aid of the shreds of cotton it passes readily to the point. Should any portion of the material pass beyond the point, it will do no harm, it may cause slight pain without any bad results. Indeed, I find it desirable in some cases of alveolar abscess, as the chloride will destroy the diseased condition, and effect a cure when other remedies fail. I recall a case where there was a fistulous opening on both sides of a lower molar which had resisted all treatment. In sheer desperation I filled the roots with oxychloride, forcing it through the point as much as possible, and filled the tooth immediately. The abscess healed in a few days, without any further trouble. Another case,—a young lady aged seventeen, who had nine dead teeth, five of them being contiguous, I treated and filled the roots as above described, the crowns with gold, all within two weeks, with complete success. Under the old system of long treatment I might not have succeeded half so well. Imagine this young lady with nine sewerage pipes in her mouth!

There is no operation more trying to the dentist and patient than that of treating day after day and week after week dead teeth as is sometimes done. The more thoroughly we clean them and the sooner we fill them the better and more successful cases we will have to report.

